# Complete Genome Sequence of a Bell Pepper Endornavirus Isolate from Canada

**DOI:** 10.1128/genomeA.00905-15

**Published:** 2015-08-20

**Authors:** Bin Chen, Mark Bernards, Aiming Wang

**Affiliations:** a Southern Crop Protection and Food Research Centre, Agriculture and Agri-Food Canada, London, Ontario, Canada; b Department of Biology, Western University, London, Ontario, Canada

## Abstract

Bell pepper endornavirus (BPEV) is a double-stranded RNA virus infecting economically important crops, such as peppers. Next-generation sequencing of small RNAs extracted from the leaves of a pepper plant showing mild viral symptoms, along with subsequent analysis, identified BPEV. The complete genome of this isolate was cloned and sequenced.

## GENOME ANNOUNCEMENT

Endornaviruses belong to *Endornavirus*, a young genus in the family *Endornaviridae* (1). Different from most other viruses, endornaviruses have several special features. They have a wide host range, including plants, fungi, and oomycetes (2). The viral genome is composed of a single double-stranded RNA (dsRNA), making it ideal for studying how they manage to evade the host RNA silencing system. Endornaviruses do not form true virions and are usually present at a low copy number (3). Plants infected by this group of viruses do not show typical viral symptoms and thus are phenotypically normal (4). Endornaviruses can infect many economically important crops, such as peppers, cereals, beans, and cucurbits and are seed transmitted (4). Bell pepper endornavirus (BPEV) is a member of the endornaviruses. The virus has spread widely in bell pepper-producing regions (5, 6). To date, the genomes of five BPEV isolates have been completely sequenced, i.e., Maor (GenBank accession no. KP455654) from the United States, YW (GenBank accession no. JN019858; also GenBank accession no. NC_015781) from the United States, IS (GenBank accession no. JQ951943) from Israel, lj (GenBank accession no. KF709944) from China, and an unnamed isolate (GenBank accession no. AB597230) from Japan. Here, we report the cloning and sequencing of the complete genome sequence of a BPEV isolate from Canada that is associated with viral symptoms.

Small RNAs were extracted from the leaves of a greenhouse pepper (cultivar Healey) showing mild crinkling and chlorosis symptoms on young leaves with the mirPremier microRNA isolation kit (Sigma-Aldrich, St. Louis, MO). Small RNA libraries were constructed with the TruSeq small RNA sample prep kit (Illumina, San Diego, CA), according to the supplier’s instructions, and sequenced using the MiSeq desktop sequencer (Illumina) with the MiSeq version 2 reagent 50-cycle paired-end (PE) kit (Illumina). After the removal of adapter sequences, the clean reads were assembled into large contigs. Searches against the NCBI database using the assembled contigs hit the genome sequence of BPEV with high (>98%) sequence similarity. Based on the contig sequences, primers were designed to clone the nearly full-length cDNA of this Canadian isolate. The 5′ and 3′ end sequences were obtained by rapid amplification of cDNA ends (RACE). The genome sequence of the Canadian isolate was determined to be 14,726 nucleotides (nt) in length. Like other endornaviruses, the genome of this isolate has a single open reading frame encoding a polyprotein containing viral methyltransferase (MTR), RNA helicase 1 (Hel-1), UDP-glucose-glycosyltransferase (UGT), and RNA-dependent RNA polymerase (RdRp). A comparison of the genome sequences revealed that the Canadian isolate shares sequence identity to the other five isolates in a range of 88 to 99% at the nucleotide level and 92 to 99% at the amino acid level. Phylogenetic analysis using the RdRp sequences was conducted. The results showed that this Canadian isolate is distinct from the YW isolate from the United States and the remaining four isolates from four different countries, which form a phylogenetic group. Taken together, we identified and determined the genomic sequence of a BPEV isolate that apparently has ability to induce typical viral symptoms in peppers.

### Nucleotide sequence accession number.

The genome sequence of the isolate described in this work has been deposited in GenBank under the accession no. KT149366.

## References

[1] King AMQ, Lefkowitz E, Adams MJ, Carstens EB 2011 Virus taxonomy: ninth report of the International Committee on Taxonomy of Viruses. Elsevier, London, United Kingdom.

[2] Okada R, Kiyota E, Moriyama H, Fukuhara T, Vaverde RA 2014 A new endornavirus species infecting Malabar spinach (*Basella alba* L.). Arch Virol 159:807–809. doi:10.1007/s00705-013-1875-4. 24122112

[3] Okada R, Yong CK, Valverde RA, Sabanadzovic S, Aoki N, Hotate S, Kiyota E, Moriyama H, Fukuhara T 2013 Molecular characterization of two evolutionarily distinct endornaviruses co-infecting common bean (*Phaseolus vulgaris*). J Gen Virol 94:220–229. doi:10.1099/vir.0.044487-0. 23015743

[4] Song D, Cho WK, Park S-H, Jo Y, Kim K-H 2013 Evolution of and horizontal gene transfer in the *Endornavirus* genus. PLoS One 8:e64270. doi:10.1371/journal.pone.0064270. PMC3647011 23667703

[5] Okada R, Kiyota E, Sabanadzovic S, Moriyama H, Fukuhara T, Saha P, Roossinck MJ, Severin A, Valverde RA 2011 Bell pepper endornavirus: molecular and biological properties, and occurrence in the genus *Capsicum*. J Gen Virol 92:2664–2673. doi:10.1099/vir.0.034686-0. 21775578

[6] Jo Y, Choi H, Cho WK 2015 *De novo* assembly of a bell pepper endornavirus genome sequence using RNA sequencing data. Genome Announc 3(2):e00061-15. doi:10.1128/genomeA.00061-15. 25792042 PMC4395069

